# Student Acceptance of Using Augmented Reality Applications for Learning in Pharmacy: A Pilot Study

**DOI:** 10.3390/pharmacy8030122

**Published:** 2020-07-21

**Authors:** Saad Salem, Joyce Cooper, Jennifer Schneider, Hayley Croft, Irene Munro

**Affiliations:** School of Biomedical Sciences & Pharmacy, University of Newcastle, Callaghan NSW 2308, Australia; Saad.Salem@newcastle.edu.au (S.S.); Joyce.Cooper@newcastle.edu.au (J.C.); jennifer.schneider@newcastle.edu.au (J.S.); Hayley.Croft@newcastle.edu.au (H.C.)

**Keywords:** augmented reality, teaching and learning, contraceptive medicines, pharmacy, simulation

## Abstract

Creating engaging learning experiences that are easy to use and support the different learning requirements of university students is challenging. However, improvements in simulation technologies, such as augmented reality (AR) and virtual reality (VR), are making such changes possible. The aim of this study is to use a mobile-based AR technology to develop an interactive learning module about contraceptive devices and medicines and to measure its acceptability and usability by undergraduate pharmacy students. The learning module comprising AR images of contraceptive medicines, case studies relating to their use and a series of directed questions was completed by 33 pharmacy students. Students answered a survey to collect information about the usability and acceptability of AR for learning. The results show that the majority of students reported that AR is a useful resource for learning about medicines compared to more traditional methods, such as didactic lectures and tutorials. Students indicated that the AR application was easy to use and improved their knowledge of medicines. These findings suggest that AR technology is a useful tool to create engaging and easy to use learning experiences for university students.

## 1. Introduction

Progressive improvements in technology are revolutionising the way educators deliver teaching while simultaneously supporting student learning. Students today have grown up with many technological advancements compared to previous generations and accept these new technologies as a preferred choice for learning [1]. Research has also shown that student learning and achievement can be enriched and improved with the use of computer technology within the classroom [2].

As mobile technologies such as laptop computers, tablets and smartphones have developed, they have become more affordable, with educational software/programs becoming more user-friendly and familiar thus enabling increasing engagement in learning using different approaches [3]. In the university sector, these learning technologies appear to be used in two ways. Firstly, institutionally supported technology, with online learning management systems such as Blackboard, available generally to all students. This technology appears to be primarily used for the delivery of learning materials for a whole course and for administration purposes [4,5]. Secondly, the use of academic-led learning technologies such as simulation, virtual reality (VR) and augmented reality (AR), which appear to be used in individual lectures tutorials and workshops within a course in specific disciplines [6,7].

Simulation uses computer technology to develop scenarios that imitate the real world while creating safe learning environments for students [8], for example, flight simulation to train pilots in aviation, and/or interactions with patients in health [9]. The use of simulation for learning has become increasingly prevalent in health disciplines with the development of novel teaching methods including computer-generated digital patients or mannequins. In nursing, for example, these simulated patients enable students to develop skills, gain experience and become competent with repeated practice, if needed, while protecting real patients from unnecessary risks [9,10]. Computer-generated digital patients have now evolved to include facial expressions and text-to-speech to provide responses that enable real-life-like interactions [11]. For example, in pharmacy, students can use the simulated patients to practice and improve their communication, diagnostic and management skills in readiness for counselling real patients [11]. Simulation instructional design features for skills outcomes such as repetitive practice, interactivity, individualised learning and feedback, have all been shown to be effective in simulation-based education [12]. Despite the many benefits associated with the use of simulation for learning, it can be costly to develop, which limits its availability and use. 

VR is an environment created by the use of computer technology that immerses participants within a virtual environment that is distinct from their physical reality [13]. It is viewed through a head-mounted display or with a series of room projectors displaying images on the walls of the room [14]. AR is a technology which uses a combination of both real and virtual images [15]. It superimposes computer-assisted virtual objects such as pictures, text or videos into the physical real world, enabling the user to interact with these images in a real situation [16]. As AR enables the user to see the real world, it supplements reality rather than completely replacing it, and it can provide a realistic learning experience for students [17]. It is suggested that using AR for learning enables students to have an absorbing and engaging environment without the loss of the authenticity of the real world [18].

The uses of VR in learning are broad and include engineering [19], education [20,21], biology [22] and medicine [23,24], whereas learning with AR has been highlighted in anatomy [25], environmental science [18], nursing [26], dentistry [27,28], and health [29]. The introduction and uptake of AR in teaching and learning has been much slower than VR. However, with the rapid developments in hand-held devices and subsequent ease of use, the application of AR has become more widespread [15]. An additional advantage of AR is that it creates a student-centred approach to learning. The design and predicted learning effectiveness of AR is underpinned by constructivist learning theory and situated learning theory [30,31]. When using AR, the learner is involved in active learning and experiential ways, which enables the student to construct their own context, knowledge and understanding through reflection [30]. Situated learning theory provides theoretical foundations for the application of knowledge from the simulated learning environment to future situations that may arise in the clinical context [31]. 

When considering the use of simulation technologies for learning, it is essential to consider ease of use and the benefits to student learning in terms of content and learning outcomes. The method chosen must also be usable and acceptable to students. Previous studies involving undergraduate science students and pre-service teachers, using AR with the software Aurasma (now HP Reveal^®^) for learning, reported that the majority of students rated the experience as positive/extremely positive and/or easy to use [32,33].

In pharmacy education, an essential area of study involves learning about medicines. The provision of authentic hands-on medicine learning experiences for students largely occurs during clinical placements; however, directing the focus of this education may be variable depending on the clinical setting. The opportunity to handle and inspect proprietary medicines in an education setting has limitations. In the classroom, it creates the challenge of maintaining an up-to-date stock of medicines outside of approved pharmacy premises, which include the legal requirements around appropriate storage and safe access for handling, in addition to high annual replacement costs. To overcome these limitations, visual simulation of actual medicines can be used to provide an augmented teaching and learning experience. This would also facilitate consistency in education for students. 

Research on the use and outcomes of AR for teaching and learning in pharmacy programs is lacking. To investigate the use of AR in pharmacy education, we are developing resources using real and 3D digital elements to create an in-house virtual library of medicines. These resources are being used to develop learning modules that undergraduate pharmacy students can also access remotely using a mobile device. Adapting the educational content in this way will enable students to access the information from both within and outside the classroom and extend their learning opportunities. The aim of this study is to use AR to develop a module on medicines and to assess student satisfaction and preference for AR for learning. The study was approved by the Human Research Ethics Committee at the University of Newcastle, Australia, approval H-2013-0151.

## 2. Methods

### 2.1. Development of the HP Reveal^®^ AR Module

The AR learning module was created and embedded as a non-assessable supplementary learning module in a Clinical Pharmacotherapy course in the undergraduate pharmacy curriculum. This course is offered over 10 weeks to third year students in the 4-year Bachelor of Pharmacy (Hons) degree at the University of Newcastle, Australia. The AR learning module was designed to provide a real-life, interactive, student-centred experience to supplement learning in the field of women’s health, with a focus on contraception and hormone replacement therapy (HRT). This area was chosen due to the complexity and availability of a vast range of medicines and proprietary brands available, which can impact the choice and the specific information a pharmacist would need to know and provide to a patient. The module consisted of two parts. The first part contained information relating to proprietary medicines, while the second part was a learning resource containing case studies to reinforce the learning from Part 1.

#### 2.1.1. Part 1: Developing the AR proprietary Medicine Resource

Seven proprietary contraceptive and HRT medicine types were obtained. These were a monophasic low-dose, standard-dose and high-dose combined oral contraceptive pill (COCP), multiphasic COCP, progesterone-containing intrauterine device (IUD), continuous HRT and cyclical HRT pills. For each medicine, photographs of the front and back of the medication box (known as the trigger images) and the contents (known as the overlay images) were taken using a *single-lens reflex* (SLR) *camera (Nikon 1)* and saved onto a standard PC as jpeg images. Using the HP Reveal^®^ online studio (Hewlett-Packard^®^, CA, USA), one trigger image jpeg at a time was uploaded to the HP Reveal^®^ site and, for each trigger image, the corresponding overlay image was then uploaded and linked. When all of the trigger and overlay images were uploaded, the resource was published on the HP Reveal^®^ site. The trigger images, size 16 × 10cm, were printed in colour on individual A4 pages. The HP Reveal^®^ app was downloaded onto an iOS device (iPad) and an Android device (Samsung tablet), and the trigger images were then scanned using the app to test that the overlay images appeared as designed, and adjustments were made where necessary to ensure that the overlay was easily triggered. The next step was to combine the images in a document and, for each image of a medicine, there were three to five questions to guide active student learning. A list of questions, relevant to different medicines, is shown in Table 1. 

#### 2.1.2. Part 2: Developing the AR Resource Case Studies

The case study trigger images consisted of cartoon images which included a picture of a hypothetical patient, a pharmacist counselling a patient in a pharmacy and a diagram of the menstrual cycle. The corresponding overlay images were either another image, an audio file to listen to or a video to watch. Audio files were recorded and saved in mp3 format, while videos were saved in mp4 format. As with Part 1 of the AR resource, the trigger images were individually uploaded and linked to the corresponding overlay files, then developed in HP Reveal^®^ online studio, printed in colour on separate pages and tested for the satisfactory triggering of overlays. Once satisfactory triggering of each overlay was achieved, the case study trigger pictures were ready to be added to the document. 

#### 2.1.3. The AR Learning Module

**Figure 1:**
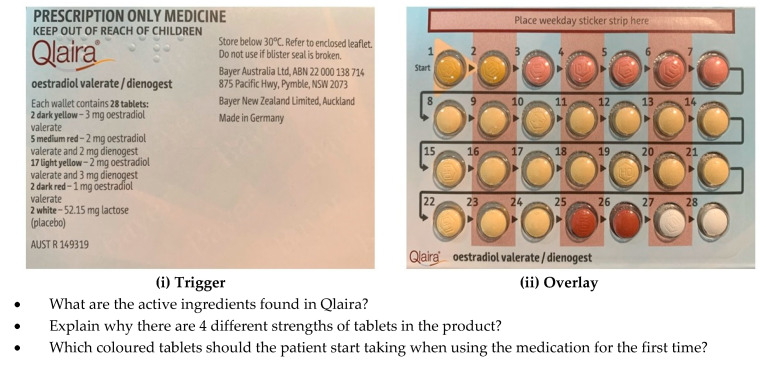
Example the trigger and overlay of a contraceptive medicine for learning using AR.

**Figure 2:**
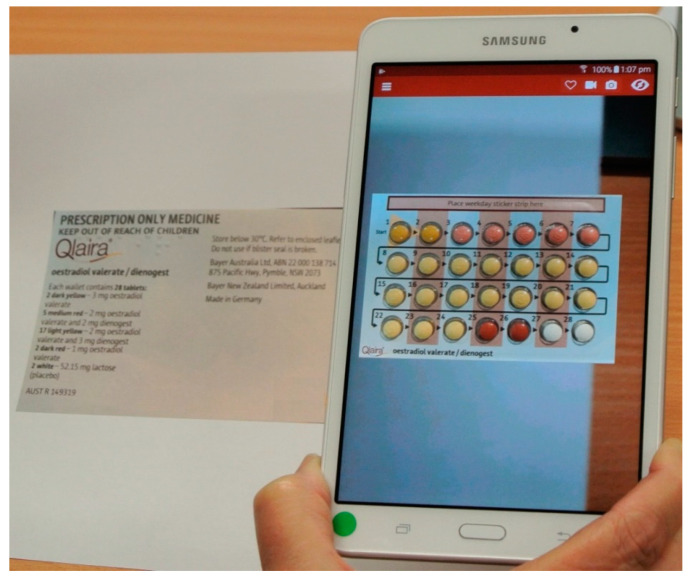
The overlay appears on a tablet when it is held over the trigger image.

The AR document included an introductory section explaining to students how to download and use the app to access the resource and how to proceed through the learning module. The first part of the module contained images of the medicines for the students to scan. Examples of a trigger image, overlay and the questions relating to that medicine are shown in Figure 1, and how the overlay appears on a tablet when the tablet is held over the trigger image is shown in Figure 2.

The second part of the module contained the case studies, to enable students to apply their learning. An example of a case study is scanning the trigger image of a patient consulting a pharmacist. This triggers an overlay of an audio of the patient asking for advice about a contraception medication. The student can then scan an image of the medication box, which triggers an image of the medicine with information about the product, which enables them to formulate the advice they would provide to the patient. 

### 2.2. Use of AR Resources in Classroom

The AR resources were introduced to the students in a face-to-face classroom setting. The students were informed how to use the software and then provided with a tablet onto which HP Reveal^®^ had been downloaded, or they were assisted with downloading the app on to their own phone. Students then progressed through the learning module at their own pace. They were able to return to previous images if they wanted to confirm or compare information about the medicines and could pause and restart or even replay the case studies. Upon completion of the module, students were invited to complete a questionnaire on the ease of use and acceptability of the AR learning module (Table 2). The beginning of the questionnaire included five demographic questions to identify any differences between the participants that might influence the results. When all students had completed the study, the AR materials were uploaded to the university’s learning management system (LMS), enabling all students to review/access the materials for personal study at any time. Statistical analysis of data was conducted using SPSS (IBM Corp. Released 2017. IBM SPSS Statistics for Windows, Version 25.0. Armonk, NY, USA).

## 3. Results

Out of the 37 students enrolled in the third-year clinical pharmacotherapy course, a total of 33 (89.2%) pharmacy students consented to participate in the AR learning experience and answered a survey questionnaire on completion. Responses to the demographic questions showed that participants comprised 33% male (n = 11) and 67% female students (n = 22). The majority of the students (84.8%, n = 28) spoke English as their first language, compared to 15.2% (n = 5) who spoke a language other than English at home. All participants (100%, n = 33) indicated that they spent 5 h or less using information technology applications on a daily basis; 51.5% (n = 17) claimed to be confident/extremely confident using AR, 33% (n = 11) were moderately confident and 15.1% (n = 5) claimed that they were not confident using AR.

On a Likert Scale of 1 (strongly disagree) to 5 (strongly agree), students rated their satisfaction and preferences for using AR for learning, as shown in Table 2. The majority of students agreed or strongly agreed that the AR learning module motivated them to learn about medicines (84.8%, n = 28) and that AR is successful at presenting medicine-related information for learning (81.8%, n = 27). Similarly, the majority of students agreed or strongly agreed that the AR app is a useful learning resource when compared to familiar methods such as lectures and tutorials (78.8%, n = 26), that the AR learning experience improved their medicine knowledge (78.8%, n = 26), and that they did not find that the AR images/videos distracted them from identifying medication-related information (67%, n = 22). The majority of students also agreed or strongly agreed that the AR app was useful for medicine training (78.8%, n = 26). Only two or three students disagreed or strongly disagreed with the statements about their satisfaction and preferences for using AR for learning, with between four and eight students remaining neutral. As the results in Table 2 show, the median score for each of the six questions was 4. The results for males and females were compared for all outcomes but were not statistically different. 

Principal components analysis shows all six questions form one factor, independent of sex. Added together, they form a scale of general satisfaction with using AR for learning, µ = 23.8, *sd* = 3.45. The scale is highly reliable (Cronbach’s alpha = 0.822.)

Two open-ended questions were included in the survey. The first question asked the students what they liked or did not like about AR for medicine learning. Thirty-two students responded to this question, with 14 students making more than one comment. Altogether, there were 49 responses. Thematic analysis was used to identify common themes that aligned with the aims of the study. Three recurring themes emerged.

Theme 1 described how students perceived their learning experiences. Descriptions included *‘interactive, active, stimulating, interesting, motivating’* and *‘visual learning helps recall’*.

Theme 2 related to the usability of medicines information. Comments included *‘Viewing information on the medicine box and the content inside at the same time made the experience real’*, and *‘concise but informative’*, and *‘conveyed really good medicine information’*.

Theme 3 focused on the ease of access to the medicines information and application of the information. Comments included *‘Everyone could access medicines at the same time’* and *‘Could be used to aid patient counselling’* and *‘Enables immediate application’*.

A fourth ‘Miscellaneous’ category was created for two responses that did not fit into any of the themes. These were *‘Eco friendly (no paper)’* and *‘Do not like AR, using scanned photos is a waste of time’*.

**Figure 3:**
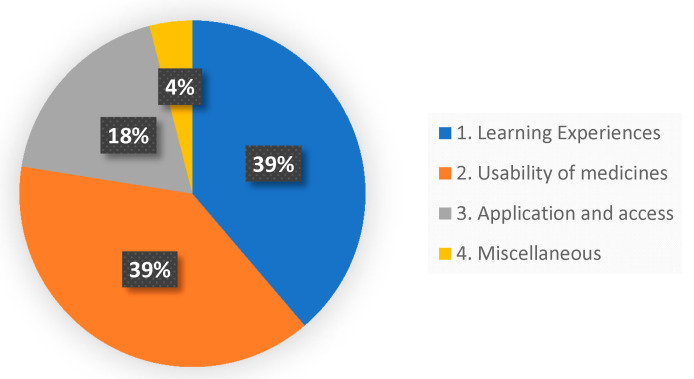
Frequency distribution of student responses by theme. (Some respondents made more than one comment: 1 comment n = 18; 2 comments n = 11; 3 comments n = 3).

The distribution of student responses is shown in Figure 3.

The second question invited suggestions on how to improve the AR app for future learning. Suggestions were made by fourteen students. Analysis of the suggestions identified two recurring themes, which included technological improvements and educational improvements.

Technological Improvements comprised nine suggestions, which included *‘vary speed of videos’* and *‘being able to pause videos’* and *‘include a function to save information’*. 

Educational Improvements comprised five suggestions, which included *‘Provide answers for the quiz at the end of the module’* and *‘Display front, back and ingredients on medication when medication is displayed’*.

## 4. Discussion

An aim of this study was to develop a student-centred learning module for a group of medicines that can be accessed in-class and on-line, and that provides a realistic medicine-handling experience, and to assess student acceptability of this module. This was driven by a need to enable students to have access to medicines beyond formal class times and extend their opportunities for learning and being work-ready. Currently, there are limitations on student medicine-handling experiences which are largely centred around experiential placements, and therefore there is variability in exposure and consistency among students. In addition, there are several factors that limit the range and handling of medicines by students within the classroom environment. These limitations include financial considerations, including the need to have a number of the same item in a classroom, particularly sufficient unit numbers of high-cost medicines; legislative factors requiring a locked safe to store drugs of dependence; safety factors, especially when storing, handling and distributing cytotoxic and refrigerated medicines, for example; and time and labour constraints, as regular stock-take and cataloguing is required to maintain currency of medicines. These all impact the use of medicines in the classroom and the creation of an authentic learning experience. 

Another issue is that, while enrolled in a course, students may have in-class access to the medicines relevant to that course, however, when the course is completed, access to those medicines is usually no longer available. Therefore, for the revision or learning and application in other contexts in other pharmacy courses, access to relevant medicines is lost. Students who are employed to work casually in pharmacies outside of university may have an advantage and can gain experience in the handling of medicines to build on the knowledge gained in class, but not all students have the opportunity to take up casual work. Providing a resource containing images of medicines or learning modules which is available outside of the classroom supports student learning and engagement. 

All the students participating in the study indicated that they used simulation technologies for five hours or less per week and although the majority (84.8%) indicated that they were moderately to extremely confident using AR, five students indicated that they were not. This lack of confidence was reflected in the results for the usability and acceptability of AR for learning by the five students who recorded a neutral opinion on the usefulness of the AR app for medicine-learning. One of these students also recorded a neutral opinion on improvement of knowledge, distraction when using AR images, and its usefulness as a teaching resource, but did strongly agree that it stimulated her interest to learn about medicines. One of the students expressed a dislike of using AR for learning and said that using scanned photos is a waste of time, which possibly reflected her stated lack of confidence using AR. However, the majority of students agreed or strongly agreed that the app was useful (78.8%), stimulating (84.8%) and that it improved their learning (78.8%). 

The responses to the open-ended questions provided insight into the opinions of students who indicated that they particularly liked the interactive experience of learning with AR, as it was *interesting, stimulating and motivating*, which aided their learning. Indeed, research has shown that interactive technology can improve student motivation and influence their achievements [34]. For example, a study in South Africa by Khan (2019) compared the learning motivation of 78 health science students before and after using an AR mobile app, and reported a significant increase in student motivation after using the AR app [35]. Similar findings were reported by Chiang et al., (2014), who investigated the learning achievements and motivation of 57 elementary school students in Taiwan using a mobile AR approach. The student’s learning achievements improved and motivation increased [36]. These outcomes are in line with the constructivist approach to learning. The augmented environment creates a learner-centred environment where students construct new information based on their previous experiences and learning [32] as they individually work through the different AR scenarios. The use of AR for learning has repeatedly been shown to increase student motivation (2,30).

Students suggested improvements to the AR module for future use in medicine learning. These were categorised into two main areas: educational improvement and technological improvement. With respect to education, students requested that more information should be provided with each task to better guide learning (15.6%, n = 5), and one student suggested that a ‘save’ function should be made available for students to save their work to use for revision in the future. Suggested improvements to the technology were related to using HP Reveal^®^, which suggested the use of a more user-friendly interface where the user has more control over the displayed images and videos; for example, the inability to pause or fast-forward embedded videos (12.5%, n = 4). Embedded audio received mixed reviews, with two students wanting to see more audio content included (6%), one student indicating that the audio content was not sufficiently clear, and one saying that it was a distraction from learning. Based on this feedback, closer attention will be given to the volume and clarity of audio in future AR module development.

## 5. Limitations

The results obtained in this study are from a small sample size of 33 pharmacy students. Furthermore, the human–computer interaction in this pilot study was conducted in an informal way in an effort to mimic a real classroom environment. However, the information obtained will enable the further development of teaching and learning resources using AR technology in pharmacy courses at the tertiary level. To use this learning technology, students must have access to hand-held devices such as tablets or phones, as well as access to the internet. It should be noted that while HP Reveal^®^ was the most appropriate AR tool to use at the time of the study, with rapid changes in technology, HP Reveal^®^ has now been superseded with similar easy-to-use software that provides similar results.

## 6. Conclusions

This study demonstrated that academic staff with minimal specialised skills in AR can easily develop AR modules for use in pharmacy undergraduate pharmacotherapy teaching. When developing new technology approaches for teaching and learning, it is important to ascertain early in the process whether students would use the technology and gauge their satisfaction and opinions on the use of this technology and how this supports their learning. This information then assists the academic in determining whether further use and development of this approach is warranted. It also guides future directions in the development of these resources. The survey of participants revealed that some students may lack confidence in using AR technology, and therefore initial support should be provided to assist students who are not confident. In addition, as indicated from the participant survey, the use of technology may not appeal to some students, and different strategies should be employed in parallel with this technology to facilitate learning for these students. Our study observed that, overall, students were very positive about using AR for learning as they found it interactive, stimulating and motivating, which encouraged their learning. Students with a less favourable view of using AR indicated that technological improvements would make the AR app more user-friendly and support learning. It was interesting to note that a few students identified how AR technology could be useful to aid patient counselling on medication. Based on this feedback, academic staff decided that further development and implementation of this approach was warranted and feedback on improvements could be incorporated into future modules to enhance the student experience.

## Figures and Tables

**Table 1:** Questions to stimulate student learning of hormone replacement therapy (HRT), contraceptive pills and devices.

What Are the Active Ingredients Found in This Medication?
How many active and inactive tablets are found in this medication?
What are the indications of this medication?
How is this medication different to the one before or after it in terms of composition?
What is the strength of the active ingredients found in this medication?
When does the withdrawal bleed start if a patient is taking this medication?
Describe the size and shape of this medication or device.
Describe how this medication or device works.
What advice would you give to a patient who missed a specific tablet or number of tablets from this medication?
Explain to the patient the appropriate way of taking this medication.
Explain the menstrual cycle and how a medication works.

**Table 2:** Usability and acceptability of AR for learning (n = 33) showing Median and Inter-Quartile Range.

	Strongly Disagree	Disagree	Neutral	Agree	Strongly Agree	Mdn	IQR
	1	2	3	4	5		
The use of the AR app stimulated my interest to learn about medicines	-	-	5	21	7	4	0
The use of the AR app is a useful teaching resource when compared with other methods for medicines training such as lectures and tutorial exercises	3	6.1	12.1	51.5	27.3	4	4–5
The AR app was able to present medicines information relating to a typical patient-pharmacist communication encounter	-	-	18.2	57.6	24.3	4	0
The AR images did not distract from identifying medicines information	-	9.1	24.2	45.5	21.2	4	0
I could identify improvement in my own medicines knowledge after using this AR app	-	6.1	15.2	48.5	30.3	4	4–5
The AR app was useful for medicines training	-	3	18.2	60.6	18.2	4	0

Mdn = median; IQR = Inter-Quartile Range.
